# On the Road to Personalized Psychotherapy: A Research Agenda Based on Cognitive Behavior Therapy for Depression

**DOI:** 10.3389/fpsyt.2020.607508

**Published:** 2021-01-08

**Authors:** Marcus J. H. Huibers, Lorenzo Lorenzo-Luaces, Pim Cuijpers, Nikolaos Kazantzis

**Affiliations:** ^1^Department of Clinical Psychology, Vrije Universiteit Amsterdan (VU), University Amsterdam, Amsterdam, Netherlands; ^2^Department of Psychology, University of Pennsylvania, Philadelphia, PA, United States; ^3^Psychological and Brain Sciences, Indiana University–Bloomington, Bloomington, IN, United States; ^4^Institute for Social Neuroscience, Ivanhoe, VIC, Australia

**Keywords:** personalized medicine, cognitive behavior therapy, depression, mechanisms of change, individual differences, moderated mediation

## Abstract

In this conceptual paper, we outline the many challenges on the road to personalized psychotherapy, using the example of cognitive behavior therapy (CBT) for depression. To optimize psychotherapy for the individual patient, we need to find out how therapy works (identification of mechanisms of change) and for whom it works (identification of moderators). To date, psychotherapy research has not resulted in compelling evidence for or against common or specific factors that have been proposed as mechanisms of change. Our central proposition is that we need to combine the “how does it work?”-question with the “for whom does it work?”-question in order to advance the field. We introduce the *personalized causal pathway hypothesis* that emphasizes the links and distinction between individual patient differences, therapeutic procedures and therapy processes as a paradigm to facilitate und understand the concept of personalized psychotherapy. We review the mechanism of change literature for CBT for depression to see what we have learned so far, and describe preliminary observational evidence supporting the personalized causal pathway hypothesis. We then propose a research agenda to push the ball forward: exploratory studies into the links between individual differences, therapeutic procedures, therapy processes and outcome that constitute a potential causal pathway, making use of experience sampling, network theory, observer ratings of therapy sessions, and moderated mediation analysis; testing and isolation of CBT procedures in experiments; and testing identified causal pathways of change as part of a personalized CBT package against regular CBT, in order to advance the application of personalized psychotherapy.

## Introduction

Personalized or precision medicine has the potential to contribute greatly to the future of healthcare by delivering the most efficient patient-centered care that is acceptable both to patients and healthcare professionals (1). Personalized medicine may be broadly defined as treatment that is highly individualized for the patient based on biomarkers, processes that relate to etiology, or findings from data-driven methods. The approach has attracted considerable attention in recent years and is considered to be one of the main challenges for health care, although there is little empirical research that facilitates its application in most fields of medicine and health care.

Depression is one of those disorders for which a personalized medicine approach is still lacking (2, 3), and arguably one of the disorders that would benefit most from a personalized approach to treatment. Depression affects the lives of many and society as a whole (4–6) and is estimated by the World Health Organization (WHO) to be a leading cause of global disability (7). Treatment options such as psychotherapy and antidepressant medication (ADM) have comparable effects (8), even in severe depression (9), although the combination of psychotherapy and ADM might be somewhat more effective *on average* (10). However, ~40–50% of patients do not respond to treatment (8, 11, 12). Those that respond remain at considerable risk for future relapse (13, 14), and even after 1 year of different treatments, about one third of patients has not remitted (15). At the same time, while some patients show almost no decrease in depression during treatment, other patients demonstrate large effects (3, 16). At this point, we do not know which patients will benefit from treatment, making treatment selection largely a matter of availability or trial-and-error (17).

In this paper, we propose a research agenda that will enable the personalization of psychotherapy for depression, in order to optimize treatment outcome for the individual patient. There are largely two distinct routes to improve the effectiveness of psychotherapy: identification of mediators to find out *how they work*, and identification of predictors and moderators to find out *for whom* they work. We propose that these two research lines need to be combined to advance personalized medicine in this area. Echoing the famous words of Paul (18), arguably the biggest scientific challenge in contemporary depression outcome research is to identify the causal pathways or *mechanisms of change* that reveal *how treatments works, and for whom*. For the context of psychotherapy, we add to this, *what works, for whom*, and *under which relational contexts*, as there are other theoretically important variables within the consultation session, not limited to generic and treatment specific elements of the therapeutic relationship. Mechanisms of change in psychotherapy for example are much debated, but poorly researched (19, 20). As a result, our knowledge is limited and the field needs innovative research methods to confirm how psychotherapy brings about its established effects (21).

In line with the focus of this special issue in *Frontiers in Psychiatry*, we will focus on cognitive behavior therapy (CBT) as the most widely studied evidence-based psychotherapy for depression. According to theory, CBT works through changes in the content and processes of cognition, emotion regulation and behavior (22). A recent review (23) of *N* = 558 meta-analyses concluded that the strongest support currently exists for cognitive (*n* = 8 meta-analyses) and behavioral (*n* = 3 meta-analyses) change processes in CBT for anxiety disorders and depression; though this evidence is still emerging and many questions remain unanswered about how to tailor these processes for the individual patient. We first describe mechanisms of change in psychotherapy research and focus on CBT for depression to illustrate what we do know about moderators and mediators. Finally, we propose a research agenda to advance the research of moderators and mechanisms and promote the development of personalized psychotherapy.

## Mechanisms of Change in Psychotherapy

We recently reviewed the literature on mechanisms of change in *all forms of psychotherapy*, focusing on the common and specific factors that might explain how psychotherapy works and concluded that most studies to date are merely correlational (21). Mechanisms of change are the elements that constitute the causal pathways of psychotherapy. Understanding how therapy works will help us improve existing therapies, develop new ones, and tailor therapy to the needs of the individual. In order to establish that a mechanism or mediator (the statistical proxy for a mechanism) is indeed a *causal factor* in the recovery process of a patient, studies have to meet several methodological criteria as previously outlined by Kazdin (19). They include temporal precedence, plausibility, experimental manipulation, consistency, association, dose-response relation, and specificity.

In our review of psychotherapy process research, none of the common or specific factors we reviewed met the threshold and can thus be considered an empirically validated working mechanism, though this research has begun (23). More than 30 years after the introduction of mediation analysis (24), we still do not have compelling evidence for the common or specific factors that bring about change in psychotherapy (25). Moreover, by definition, psychotherapy is a complex process that involves multiple factors, dichotomies of common vs. specific factors are questionable (26), and simple causal models will not advance our understanding of the underlying mechanisms of change.

Our review (and previous overviews) also revealed that mechanism research is very challenging, and that most previous studies suffer from methodological shortcomings that limit the usefulness of findings. We have summarized the most important methodological problems and opportunities (21, 27, 28):

1. **Most mechanistic research has been conducted within the context of a randomized trial**. More experimental studies in which the proposed *mechanism* (as opposed to the *intervention*) is directly manipulated would be more informative.
2. **CBT is treated as a black box**. Its therapeutic procedures (e.g., interventions aimed at cognitive change) and the change processes (e.g., the cognitive change itself) that follow from them are rarely distinguished.
3. **Most measurement is concurrent**. Temporality in research designs is needed to *establish a time line* that shows which of the constructs change first in order to rule out *reverse causality*.
4. **Little attention has been paid to individual differences**. Data on mechanism are mostly analyzed on the average group level. It is likely that patients differ in their response to therapeutic procedures offered to them, and these variations should be taken into account (i.e., *mediation moderated* by patient characteristics).
5. **Most previous studies relied on older approaches to mediation testing** (24). More modern approaches (29, 30) are more flexible and have more statistical power.

As a result, *how* CBT (or any other form of psychotherapy) works is still a ***black box*, **as was recently described in terms of a **personalized causal pathway hypothesis** (31).

The central proposition here is that therapeutic procedures (e.g., how therapeutic techniques are targeted and used to help patients change negative thinking) should be distinguished from (intra-individual) therapy processes (e.g., decrease in negative thinking) in order to crack the black box. Similar distinctions were proposed by Doss (32), who also underscored the importance of therapist change procedures (e.g., explaining the process to complete a thought record) and client change procedures (e.g., examining evidence for or against a belief). Moreover, mechanisms of change most likely differ between (subgroups of) individuals, and these individual differences need to be considered to unravel how psychotherapy works. It is not only about how psychotherapy works (mechanisms) but also for whom (moderators).

Moderators are prescriptive variables (i.e., patient characteristics) that predict a differential outcome in two or more treatments. Unlike general predictors (prognostic variables) they points us in the direction of the underlying mechanistic pathways that are active in specific subgroups of patients (19), without necessarily revealing what these pathways are. If certain patient characteristics predict a differential outcome depending on type of treatment, it must mean that something specific in the type of treatment is driving response in certain individuals and not in others. Further complexity exists because the clinician is tailoring therapeutic procedures (treatment processes) within the treatment and their mode of delivery (in-session process) as a function of patient characteristics (33). Thus, finding moderation is ipso facto evidence of *differential* mediation, i.e., pathways of change that differ between two or more treatments. It also implies that mediation analyses should take individual differences into account, since proof of moderation also means that subgroups of patients are responding differently to the mechanisms that are triggered in treatment. We will later return to this issue of *moderated mediation*.

## What Do We Know? the Example of CBT for Depression

### Cognitive Behavior Therapy and Its Putative Mechanisms

Of all psychotherapies for depression, ***cognitive (behavior) therapy***(CBT) is the most extensively researched (34, 35). CBT is an effective treatment in the acute phase of depression (36), can prevent future relapse (37, 38) and is a recommended choice of treatment in clinical guidelines (39).

According to Beck's cognitive theory (40), dysfunctional beliefs about the self, the personal world and the future, incorporated in stable and enduring schemas, lie at the root of depression. When activated by stressful events, these (implicit) schemas produce negative thoughts and depressive symptoms.

CBT aims to change negative thinking and alter dysfunctional behavior, by restructuring thoughts and increasing physical activity. Central to CBT is the assumption that *cognitive change* is the mechanism that leads to recovery in CBT (41). If cognitive change *is* the central mechanism in CBT, how exactly does it work? Barber and DeRubeis proposed three different models (42):

- ***Accommodation model****:* CBT changes (explicit) negative thoughts and (implicit) underlying schemas directly, in such a profound way that the individual's depressive symptoms, and the risk for relapse, are reduced.
- ***Activation-deactivation model***: CBT merely deactivates (implicit) underlying schemas temporarily, leaving the underlying vulnerability for future depressive episodes untouched.
- ***Compensatory skills model***: CBT leaves the (implicit) underlying schemas unchanged, but promotes the use of certain compensatory skills for dealing with distressing thoughts and events.

The empirical support for any of these models is weak, as only a handful of studies provide preliminary, typically indirect evidence (28, 41), though cognitive change is the best “contender” for a working mechanism of CBT. Tang and DeRubeis (43) found so-called “sudden gains” in CBT, sudden improvements in depression following substantial change in negative thinking in the *preceding* therapy session, which indicates that cognitive change might drive the observed improvement. Dozois et al. found that CBT was associated with greater change in schemas than antidepressants (44). Schmidt et al. (45) applied a fine-grained session-to-session analysis to demonstrate that the relation between immediate cognitive change in a previous CBT session and subsequent depression change in a following session was mediated by the sustained cognitive change measured at the beginning of the following session. Moreover, both immediate and sustained cognitive change predicted subsequent symptom change, and the only variable that predicted immediate cognitive change was therapist adherence to cognitive methods. This not only reveals that cognitive change may be a predictor of symptom change, but also highlights cognitive change as a potentially important mechanism of change, at least in CBT.

Other studies found that change in negative thinking is *not specific* to CBT, and can also be observed in other psychotherapies and antidepressant treatment (41). However, the question of whether measures are sufficiently specific to target the spectrum of change in cognitive content and process (e.g., attentional refocusing, beliefs about intrusive thoughts and ruminative processes) and the concomitant comprehensiveness of the assessment strategy remains a matter for debate. For example, acquiring certain skills as a result of therapy (e.g., examining one's own thoughts) has been linked to symptom decreases and relapse prevention after therapy (46, 47), yet patients are likely to develop different beliefs about their thought processes through this work that were not assessed. While promising, most of these findings are merely *observational* and do not provide strong support for causal inferences, and also are technically unable to tap the full spectrum of changes that each patient experiences as they benefit from therapy. *Experimental studies* in which a putative mechanism is manipulated to provide a direct test of causality are almost completely lacking, with the exception of an older study (48) in which it was found that attempts to *change* thinking processes led to a greater reduction in negative thoughts and depressive symptoms, relative to efforts to *explore* thinking processes.

In recent years, treatments that focus solely on behaviors have received renewed attention. *Behavior therapy* was developed in the 1950's, but was overshadowed by the “cognitive revolution” in the 1970's that followed from the work of Beck and others (49). However, findings suggest that *behavioral activation* (BA, the behavioral component of CBT) *alone* is as effective as a full package of CBT (50–52). A heated debate on what these results tell us about the underlying mechanisms of change continues to this day. Some have argued that the equivalence of BA and CBT proves that both BA and CBT work only through behavioral changes (53). Others have pointed out that we cannot draw this kind of conclusion from comparative treatment studies (49).

In our view, it is still entirely possible that both BA and CBT work through changes in negative thinking, as studies with carefully planned assessments of the relevant behavioral and cognitive change processes are lacking. A further possibility is that there are other features of CBT such as empiricism that exist to a lesser extent in therapies that have a behavioral focus, or are adopted differently. If these features of CBT are not measured within trials contrasting “behavioral” and “cognitive” components, unmeasured variability within conditions could explain the comparable findings. As long as we cannot determine *which process changes precede changes in depression symptoms*, it is impossible to determine which mechanisms account for the effects of CBT (or BA), and even temporal precedence does not provide conclusive evidence that these processes are the actual cause of the change in depression.

CBT theory assumes that CBT works through *specific*, CBT-related elements. However, there is a competing model that has gained considerable popularity among therapists especially that claims that the effects of therapies are realized predominantly by so-called *common factors*. These common factors are those factors that all therapies have in common, such as the therapeutic alliance between the patient and the therapist, expectations, and a rationale that helps the patient understand his problems (21). The most modern common factors model is the *contextual model* (54), according to which a patient and a therapist first have to create a basic bond to work together. The contextual model and common factors hypotheses are supported indirectly by correlations between the therapeutic alliance and treatment outcomes, but there are no experiments that have manipulated this therapy process directly (21), there are concerns about the conduct of the meta-analyses used as support for the model (55) and serious concerns about the validity of the conceptual model across therapeutic modalities with those correlational findings (56).

A further problem is that common factors may be used in CBT in specific ways that mean they are no longer “common” and comparable to what occurs in other therapies (57). For example, understanding with empathy and interpersonal effectiveness are part of the operationalization of therapist skill in CBT delivery, as they require a specific focus on understanding the patient's cognitive internal reality in a manner that is highly professional. Yet these aspects are also part of the therapeutic alliance as conceptualized in scales such as the Working Alliance Inventory (58). Lorenzo-Luaces et al. (57) found evidence that the effect of the alliance varies by prior episodes markedly in CBT, but not in psychodynamic therapy, suggesting that this supposedly “common” therapy element may operate in different ways across different treatment. Evidently, there is a need to reliably assess modality specific elements of the therapeutic interaction, determine if they predict CBT outcomes over and above the effects of common factors, and then conduct experimental studies where they are experimentally manipulated in order to validate their importance for CBT.

### Mechanisms and Moderators in CBT: Implications From the STEP-D Study

To illustrate the intrinsic links between mechanisms and moderators, we describe the results of a randomized trial comparing CBT and interpersonal psychotherapy (IPT) for depression from the first author's research group, the STEP-D study that was conducted at Maastricht University. Depressed patients seeking help (*n* = 182) at an academic mental health clinic were randomized to a maximum of 20 sessions of CBT and IPT and monitored up to 2 years after the start of the study. CBT and IPT demonstrated comparable effectiveness in the reduction of depression severity *on average* in the acute phase (59) and in the long term (60).

Using latent-difference score models, we then examined the role of five (common and specific) therapeutic processes (dysfunctional attitudes, interpersonal functioning, rumination, self-esteem, and therapeutic alliance) that were repeatedly measured during therapy as potential mediators of outcome (61). Although processes were associated with outcome and changed in the expected direction, change in processes was remarkably smaller than change in symptoms. More importantly, no *temporal relations* between processes and outcome or mediational paths were found, which led us to conclude that the theoretical models for CBT and IPT could not be confirmed.

On the other hand, we found evidence for moderation in this study, which suggests that different mechanisms are active in CBT and IPT. In one paper, we identified general baseline predictors and moderators of treatment outcome that were then combined in a so-called Personalized Advantage Index PAI (62) to determine which of two treatments is predicted to produce the best result for the individual patient (63) ^1^. Five moderators predicted a better outcome in CBT while only one moderator predicted a better outcome in IPT. A high PAI score indicates a large predicted difference in outcome between two treatments, in this case CBT or IPT, and the average PAI in our sample was 8.9 BDI-II points, with larger PAI scores for those who were predicted to do better in CBT than for those who were predicted to do better in IPT. In additional analyses, comorbid anxiety (diagnoses and symptoms) and higher cluster A/B personality traits were also associated with better acute outcomes in CBT compared to IPT (64, 65). Moreover, it was found that “sudden gains” occurred significantly more often in CBT compared to IPT (27), which also may point at differential mechanisms being active in the two psychotherapies.

Taken together, the STEP-D findings suggest that CBT and IPT may work (partly) through different mechanisms, but which mechanisms remains unclear (66). Given the many moderators predicting favorable outcome in CBT, the larger PAI scores favoring CBT and the occurrence of sudden gains, it might be implied that these mechanisms are more active and pronounced in CBT compared to IPT (at least in these data), which speaks to the specificity of CBT. There are several possible explanations why we did not find evidence of differential mediation: the statistical power was lacking, the theories might be wrong, we might have measured the wrong constructs or used the wrong methods and design. We think one important and very likely explanation is that we did not factor in individual differences, in the form of moderators. One alternative may be to use PAI scores as indices of individual differences in the relative likelihood of benefitting from the mechanisms of one treatment vs. the other. The evidence of moderation in the absence of evidence for mediation means that we now know that mechanisms exist, but that we do not know what they are or in which patients they work. Testing for *moderated mediation*, mediation moderated by patient characteristics, might then be the answer.

### Pathways and Individual Differences in CBT

**Figure 1:**
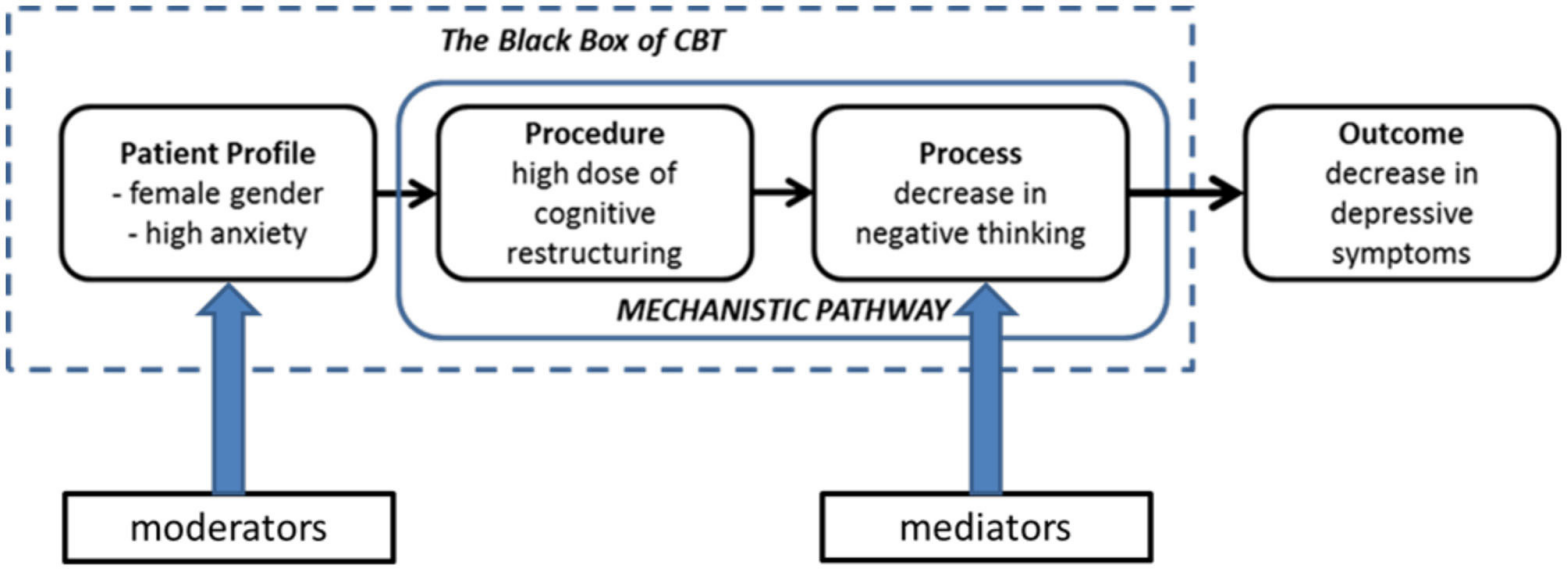
Hypothetical causal pathway linking profile-procedure-process-outcome in CBT. Procedures and processes have often been lumped together under the term “mechanism.” Note that the temporal precedence (what follows from what?) is important here.

A central proposition in this paper is that we should break down the elements that constitute the (potentially causal) pathways of a CBT intervention in order to understand what is inside the black box. We should distinguish certain individual ***patient profiles***or subgroups that are associated with individual differences in outcomes and processes of therapy, therapeutic ***procedures***that are applied in therapy, therapy ***processes***that follow from the procedures applied, and ***outcome***in terms of depressive symptomatology (Figure 1).

Is there evidence to support this ***personalized causal pathway hypothesis***? Recent observational CBT findings (Table 1) seem to point in this direction. Lorenzo-Luaces et al. (67) found that for individuals with fewer than three prior episodes of depression there was a moderate correlation between *observer-rated* therapeutic alliance (a process) and outcome, whereas there was essentially no relation in the subgroup of patients with three or more episodes. This pattern of results was replicated in the CBT condition of another RCT, but not in psychodynamic therapy (57). This pattern of results suggests that specific (e.g., CBT vs. PDT) and “common” therapy factors (e.g., working alliance) interact to predict outcomes. In an analysis of the relationship between therapist adherence to cognitive therapy methods and symptom changes in a depressed sample, Sasso et al. (69) found that cognitive methods were more strongly related to next session change for women compared to men. In addition, fewer prior depressive episodes and higher pre-treatment anxiety predicted stronger relationships between use of behavioral methods and change in symptoms. Similarly, Webb et al. (70) found evidence that therapist adherence to CBT techniques was most strongly associated with response among individuals with more severe depression. Keefe et al. (71) found that depressed patients with personality disorders benefitted from a therapeutic focus on maladaptive core beliefs but did not experience benefit from other procedures.

**Table 1:** Overview of identified links between profiles, procedures, processes, and outcome.

**Study**	**Profile**	**Procedure**	**Process**	**Outcome**
Lorenzo-Luaces et al. (57)	Prior episodes	CBT vs. PDT	Alliance	Symptoms
Lorenzo-Luaces et al. (67)	Prior episodes	n.a.	Alliance	Symptoms
Zilcha-Mano et al. (68)	Not intrusive, but cold	n.a.	Alliance	Symptoms
Sasso et al. (69)	Women	CBT methods	n.a.	Symptoms
Webb et al. (70)	Severity	Adherence	n.a.	Symptoms
Keefe et al. (71)	Personality disorder	Schema work	n.a.	Symptoms
Forand et al. (72)	“Moderate” prognosis	Engagement/adherence	n.a.	Symptoms

Moreover, there is also evidence that multiple patient variables may interact to predict outcomes. Utilizing a data-driven approach, Zilcha-Mano et al. (68) used machine learning techniques to identify patient characteristics that moderate the alliance-outcome association. They found this association to be strongest in a subgroup of patients “rated as not overly intrusive but who were overly cold in their affect toward others.” This suggests that research may need to move beyond considering isolated variables.

Using data from an earlier RCT on web-based CBT for depression (73), Forand et al. (72) tested the *prognosis moderation hypothesis*, which states that patients with a “moderate” prognosis will evidence stronger process-outcome relationships than patients with a “good” or “poor” prognosis (56). Specifically, they used multiple patient pre-treatment variables to create a prognostic index. Results showed that those in the “good prognosis” group improved regardless of the therapy procedures received while those in the “poor prognosis” group remained depressed and were not affected by therapy procedures. Conversely, for patients with a moderate prognosis, there was an association between adherence to the intervention and treatment outcomes, in line with the prognosis moderation hypothesis.

All of these studies are *post-hoc* analyses with a limited power and publication bias cannot be ruled out here, but the findings might form bits and pieces of a promising puzzle regarding the mechanisms of change in CBT. However, studies **directly** linking patient profiles, exact procedures, processes, and outcome are still lacking to this day, and there is no unifying account of change in CBT.

## What Do We Need? A Research Agenda

In this section, we describe three related research objectives that will help to discover the mechanisms of change that are active in psychotherapy and that should be targeted in a sequential order, by which each step informs the next step: the identification of mechanisms using large observational datasets, the experimental isolation of specific therapy procedures to assess their effect on processes and outcome and the development and testing of personalized psychotherapy packages. We continue to use CBT for depression as an example, but this framework can also be applied to other forms of psychotherapy.

### Identification of Mechanisms

Most process research essentially demonstrates how difficult it is to determine the processes that account for outcomes in psychotherapy, mainly because the research design falls short (25). One likely reason is that the utility of questionnaires to capture process changes is limited (28), while day-to-day assessments of single symptoms and processes might be more appropriate to capture the fluctuations of mood and mind states. Moreover, we have failed to distinguish therapeutic procedures and subsequent therapy processes in a clear way, although more recent work has begun to disentangle these related but distinct phenomena (28, 33, 74).

How *can* we open up the black box of CBT? We propose an exploratory study framework that combines daily assessments of relevant constructs (“experience sampling”) and observer-rated assessments of procedures during the course of CBT to establish the (potentially causal) links between therapeutic procedures, therapy processes and subsequent outcome, and investigate whether these links differ in subgroups of patients. DeRubeis et al. (56) noted that for some subgroups of patients the therapy procedures they receive will have a greater impact on outcome. They hypothesize that patients who are pre-disposed to have a favorable prognosis, will obtain positive outcomes regardless of the quality of therapy they receive, while another group of patients will not respond to therapy regardless of its quality. This supposes the existence of a third group of patients who will only respond to therapy if the quality is sufficient. In this latter group, therapy procedures will most likely be related to outcomes, but process-outcome associations for this subgroup are lost in the aggregate of (trial) data. The statistical concept of *moderated mediation* (75) captures the idea that there are differential mediational processes across subgroups of patients. In other words, “different folks need different strokes,” and recent studies have just begun to explore these associations (28, 67, 68).

#### Network Theory

The *network theory of psychopathology* was introduced by Borsboom and Cramer (76, 77), who state that mental disorders likely result from the causal interplay between individual symptoms that involve feedback loops, wherein symptoms fuel each other. In their terms, “causal meaningful relations are the stuff of which mental disorders are made.” They also propose that *therapeutic procedures* should be targeted at these core symptoms and the relations between them, and conclude that this approach would sit especially well with an intervention like CBT. In collaboration with the Borsboom group, we used data to characterize a network connecting depression symptoms measured before each session in the course of psychotherapy, with some symptoms being more “central” than others (78). We propose to extend this approach to link symptoms and processes. We also propose to link this network of symptoms and processes to observer-rated procedures, to determine if and how therapeutic procedures break the connections of maladaptive symptoms and processes that perpetuate depression. The advantage of the network approach is that it seeks to identify (potentially causal) *within-person* changes, whereas standard nomothetic approaches, based on between-person changes, assume that all individuals respond in the same way to therapeutic procedures. Distinguishing between-person variability (e.g., degree of negative thinking) and within-person variance (e.g., change in negative thinking over time) is of great importance to assess how changes within a patient in the course of treatment lead to individual outcomes.

Use of advanced methods such as experience sampling (ESM) might be very helpful to track down change processes of individual patients before and during treatment that can then be linked to outcome [see for e.g., Fisher (79)]. In ESM (80), participants are asked to rate their momentary experiences daily at random times, using an electronic device (i.e., smartphone). The set of single items refers to concrete experiences, such as “how sad do you feel right now?” or “how much are you bothered by negative thoughts right now?” Collected over longer time periods, ESM results in a very large number of observations per individual. The advantage of ESM is that it has a high ecological validity, takes the dynamics of daily life into account and yields high statistical power. In our example, single items to be assessed daily address depressive symptoms (e.g., sadness, guilt, restlessness, concentration), well-being, negative thinking (e.g., dysfunctional cognitions, rumination, intrusive images) behavior (e.g., activity, avoidance, use of therapy skills), and interpersonal functioning (e.g., social relations and activity).

However, ESM also comes with its challenges. The use of single items makes it difficult to account for measurement error. Moreover, the use of session-by-session assessments in combination with intensive ESM might be a burden for participants, which underlines the need to keep participants engaged in the study. ESM needs rich and dense data to robustly model time series, also because the time between sessions is rather short and violations of stationarity can become a problem. However, we think the advantages of ESM outweigh these challenges.

#### Processes to Be Investigated

As said, CBT is assumed to work through cognitive and behavioral procedures that lead to less *negative thinking* and more *positive reinforcement* and *activation*. But there are other *candidate mechanisms* too. In recent years, research has highlighted the role of *therapy skills*, i.e., skills and strategies that are acquired as a result of therapy. Barber and DeRubeis (42) proposed that these compensatory skills (defined as the ability to identify and challenge depressive, dysfunctional thoughts or beliefs) can become an automated process as a result of continued practice and might form the central process in CBT. Moreover, they suggested that either the activation of other more functional cognitions and schemas (and deactivation of the dysfunctional ones) or cognitive change could be explained as a result of the repeated use of these skills. CBT skill acquisition has been shown to be associated with greater depression reduction during therapy, as well as resistance to relapse after therapy is terminated (46, 47, 81).

Related to therapy skills are the *learning processes* that take place in therapy, particularly the role of *memory* (74). Harvey et al. (82) proposed to improve therapy outcomes by improving memory for in-session therapy information and content. We have proposed that therapy outcomes can be improved by increasing the frequency of therapy sessions (from once- to twice-weekly), with increased learning processes that lead to better skills as the underlying mechanism (83), a hypothesis we are currently testing in the context of a large randomized trial (84). Other phenomena that are relevant in this context are *mental imagery* and *rumination*. Depressed patients report having intrusive negative images about past experiences (85), and imagery is known to enhance memory (86). Rumination is defined as “repetitive thinking about the causes, meanings and implications of depressed feelings, symptoms, problems, and upsetting events” and has been shown to play an important role in depression (87, 88). Studying these cognitive processes *in conjunction* will likely advance our understanding of the mechanistic pathways that are engaged in CBT.

Finally, the *therapeutic alliance* between the patient and therapist has been championed as the essential mechanism according to the *common factor theory*, that states that a-specific elements present in all types of therapy are responsible for the effects (20, 89). Several meta-analyses show that a strong therapeutic alliance is linked to treatment success in psychotherapy, although the association is modest (90, 91). Findings like these have been presented as evidence for the common factor model, but associations cannot be used to infer causation. More recent studies (in CBT and other therapies) have tried to push the ball forward by disentangling the temporal sequence of change, but the evidence remains far from conclusive, as we reviewed (20).

To expose to the pathways that link profiles and procedures to processes and symptom change in CBT, large observational studies in depressed patients who receive CBT that is considered to be of high therapeutic quality are needed. An observational study is preferred because randomized trials are designed to diminish the individual variability we are interested in and leaves out the (potentially large) group of patients that is not willing to be randomized for treatment. First, it should be identified which processes account for recovery, and whether this differs between subgroups. Second, it should be investigated how processes and individual symptom changes are dynamically interconnected in time using network analysis (77, 78). Third, it should be investigated whether observer-rated CBT procedures can be linked to the identified processes and process-symptom connections. Fourth, the findings from these different explorative steps should be combined to *determine distinct pathways* of profiles, procedures, processes and outcome, and determine which kinds of patients need which procedures to engage which processes that drive recovery from symptoms.

#### Observer Ratings of In-session Procedures and Processes

In order to acquire observer ratings of the relevant procedures and processes, all therapy sessions in the observational study should be videotaped. Tapes can then be studied by independent raters, who rate the occurrence and magnitude of process changes and the procedures that are applied in-session, and the overall quality of therapy, using pre-defined rating scales such as Collaborative Study Psychotherapy Rating Scale (92) and the Cognitive Therapy Rating Scale (93). Because of the complexity and expert level needed to do this, procedures, processes and therapy quality should be rated by different groups of raters. Obviously, rating all sessions is a tremendous amount of work, and the method is not consistently applied in the psychotherapy literature. However, this laborious behavioral analysis method has been successfully applied in several studies (43, 94–97), as it provides the strongest test of therapy adherence, therapist competence, in-session process changes and the delivery of procedures. One alternative to make the process of rating more feasible is the use of thin-slicing or related procedures that only rate a portion of the therapy session. Another alternative is to rate the content of internet-based therapy procedures where a substantial amount of therapy content occurs via text exchanges. In a recent study, 90,000 therapy hours from 17,000 patients receiving internet-enabled CBT were analyzed using deep learning methods to associate therapist utterances with outcome. It was found that increased quantities of CBT techniques, especially CBT change methods, were positively associated with reliable improvement, while the quantity of non-therapy-related utterances was negatively associated (98). Although it is not clear whether the techniques that came up in therapy were actually delivered appropriately, the methods applied in this large-scale study might also advance our understanding how therapists' behaviors are linked to processes and outcome.

#### Hypotheses

The ***default hypothesis***that is usually tested in most studies is that CBT works through its theoretically assumed working mechanisms, namely that CBT procedures lead to changes in negative thinking and (depressive) behavior, which leads to a reduction of depression symptoms. However, the ***personalized causal pathway*** ***hypothesis***we propose states that CBT works through its theoretically assumed working mechanisms, but that causal pathways differ between subgroups of patients, and that these pathways contain interactions of procedures and processes that are more complex than traditional CBT theory states. For instance, *cognitive restructuring* may lead to *cognitive change*, but only if the *therapeutic alliance* is strong, and only if patients have a high *educational level*. The ***alternative hypothesis***is that CBT does not work through its theoretically assumed working mechanisms, but because of “common factors” present in all forms of psychotherapy, such as the therapeutic alliance.

An overview of the main points that were made in section identification of mechanisms is presented in Table 2.

**Table 2:** Main points of section identification of mechanisms of change.

**1**	**Exploratory and observational study frameworks to study the links between therapeutic procedures, therapy processes and outcome, relying on not only self-report assessments but also observer ratings of procedures and processes that are manifested in session**.
2	Careful consideration of the therapy processes that are the strongest candidates to reflect actual mechanisms of change according to theory, such as CBT processes, learning processes and the therapeutic alliance.
3	Moderated mediation analyses to find out for whom certain procedures and processes matter most.
4	Network analyses that link the (potentially causal) connections between symptom change and process change by means of rich experience sampling data.

### Isolating CBT Procedures in Experiments

**Figure 2:**
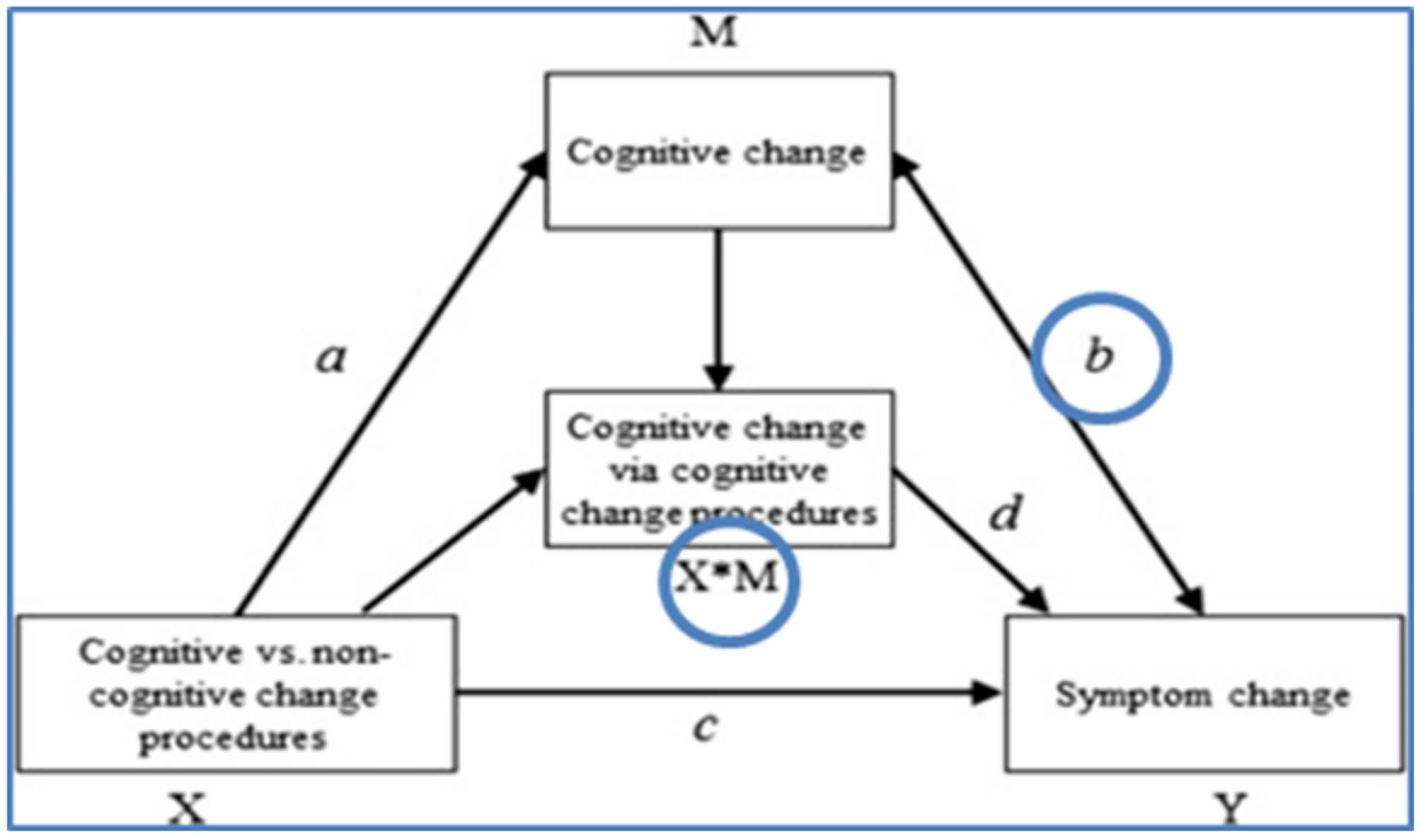
Mediation model to be tested in an experiment (28).

A much-echoed criticism regarding existing mechanisms studies is the lack of experimental designs in which the putative mechanism (or mediator, in statistical terms) is isolated and *directly* manipulated to assess a possibly *causal* effect on outcome (19, 21) (Figure 2). Here too, it is essential to distinguish procedures and processes, as sequential parts of the mechanistic pathway. The second line of research we propose is a series of experiments in which the most important CBT procedures are isolated and compared to a non-active control condition in a sample of depressed patients so that the effect on relevant processes and outcome can be assessed. For example, cognitive restructuring (using Socratic questions to evaluate negative thoughts, using dysfunctional thoughts records with guided therapist support) could be compared to merely monitoring negative thinking (using dysfunctional thoughts records), while in another experiment behavioral activation (increasing pleasurable activities, using activity registration) can be compared to merely monitoring daily activities (using activity scheduling). Study participants who experience at least mild depression symptoms would receive a series of tightly scheduled sessions (e.g., six 30-min sessions in 2 weeks, to optimize the effect of the manipulation) that focus solely on the isolated procedure. Experience sampling methods can be added in the course of the experiment to collect rich momentary data on daily experiences, processes and symptoms.

To illustrate, we describe a few preliminary experiments recently conducted at the *Experimental Psychotherapy Lab Amsterdam* in which we have begun to conduct such mechanistic experiments with (distressed) students. In the first experiment, the effects of cognitive therapy skill acquisition (*n* = 36) were compared to no procedure (*n* = 36) in response to induced distress following a social stress test. Participants reported more cognitive therapy skills after the procedure focused on the acquisition of cognitive therapy skills compared to no procedure, but there were no differences in dysfunctional thinking, distress and mood between the groups (99). In a second experiment, distressed students were randomized to an experimental condition focused on the acquisition of cognitive therapy (CT) skills (*n* = 27) or a control condition focused on being exposed to theories of automatic thinking (*n* = 25), after which all participants were exposed to a sad mood induction. Participants in the experimental group used more CT skills compared to participants in the control group, but there were no differences between conditions in the decrease of the credibility of idiosyncratic dysfunctional beliefs and strength of emotions. However, in participants with low levels of depression, those who underwent the experimental procedure showed larger decrease in the credibility of their most malleable belief (i.e., mostly automatic negative thoughts) compared to those that received the control procedure (100). In the third experiment designed to test the role of memory of therapy content, individuals with moderate levels of distress were randomized into retrieving (memory test, *n* = 46) or rehearsing (restudying, *n* = 49) four weekly sessions of online problem-solving therapy (PST). Retrieval led to overall higher recall, but this difference disappeared when controlling for the time spent on retrieval vs. rehearsal (101). Retrieval did not lead to better problem-solving skills or less distress, compared to rehearsal. Baseline working memory performance did moderate the effect of condition on recall.

Taken together, these *preliminary experimental studies* shed new light on the required dose and intensity of CBT procedures, the impact of initial depression severity, the advantages in conducting these experiments, and the importance in extending this research program to clinical populations. Using designs like these, we might be able to test the direct effects of isolated CBT procedures on therapy processes and depression symptoms in patients with depression.

### Testing Personalized CBT

Once we have collected sufficient findings along multiple, converging lines of research on the patient profiles, procedures, processes, and outcome that might constitute the individual pathways of change in CBT, we can use these new empirical insights to develop personalized CBT packages based on procedures that are deemed to be crucial for certain individuals or subgroups of patients. Of course, the ultimate experimental test to demonstrate whether these personalized pathways are truly causal in nature would be an RCT in which a personalized CBT package based on the identified pathways of change that can be matched to the individual patient outperforms a standardized CBT package, in terms of both process change and outcome.

Let's assume we have found *one* potentially causal pathway linked to a certain subgroup and that this subgroup consists of female patients with high anxiety that is predicted to respond best to a (single) CBT procedure (i.e., a high dose of cognitive restructuring) that leads to a change in process (i.e., less negative thinking) and a subsequent decrease of depressive symptoms (see Figure 1). In this simplified hypothetical example, personalized CBT would consist of a higher dose of cognitive restructuring delivered with a specific timing (e.g., in session 3–12) compared to standard CBT, and less focus on behavioral activation or other procedures, based on the potentially causal pathway we found. In case we find two or more causal pathways linked to other subgroups as well, we could include these in the trial, with the characteristics of the subgroups as additional inclusion criteria. In fact, it makes sense to compare several personalized CBT packages (that are likely to be highly variable and to contain more carefully planned interventions than in this simplified example) to standardized, one-size-fits-all CBT in a randomized trial if we want to demonstrate that selecting specific therapeutic procedures for specific patients leads to better and perhaps also faster recovery from depression.

## Conclusion

In this paper, we described the many challenges on the road to develop personalized psychotherapy that fits the needs of the individual patient and presented our ideas how to improve our understanding of the mechanisms of change in psychotherapy. We used the example of CBT for depression because it is the most extensively researched form of psychotherapy with a relatively large evidence base on outcomes, predictors and processes involved. We left out the statistical analysis considerations that come with complicated designs such as these as the field is likely to follow the rapid developments in statistical methods such as network modeling (76) and machine learning approaches (17). We also did not provide an overview of alternative designs that might be particularly useful to study mechanisms of change, such as single-case series designs (102).

The topic of our paper is a timely one, and many others are presently contributing to the debate. As part of the Lancet Psychiatry Commission on psychological treatments research, Holmes et al. (103) described the many difficulties of mechanism research, such as the lack of rigorous methodology that plagues many mediation studies. They too promote the study of moderators to improve precision in treatment matching but also to learn more about the (differential) mechanistic pathways in psychotherapy, and the “unpacking” of psychotherapy packages by focusing more on therapeutic strategies (i.e., procedures). Future studies should demonstrate whether matching mechanistically focused treatments to individual profiles enhances treatment outcome. Kazantzis (33) proposed the “matrioshka process,” a testable model of the different therapeutic techniques and in-session processes that are involved in CBT as a means to understand their true relations and provide an empirical basis to tailor therapy to a particular patient at a particular point in therapy. Hofmann and Hayes (104) have suggested that the future of intervention science should be focused on therapy processes. They state that the medical illness model of psychopathology has lost its utility (and as a result the term CBT perhaps as well), and that we should move forward toward process-based therapies that target core mediators and moderators directly based on testable theories, that link evidence-based therapeutic procedures to evidence-based processes and that are ideographic rather than nomothetic in nature, consistent with the overall trend toward more person-centered approaches. Watkins et al. (105) described an innovative study framework to distinguish therapeutic procedures and processes and investigate causal pathways of change. They propose to use (fractional) factorial designs to identify the active ingredients of internet-delivered CBT for depression, framed within the Multiphase Optimization Strategy (MOST) approach. The optimization phase is used to select the candidate components that should be included in the optimized intervention, which can then be tested against the standard intervention in the evaluation phase. This of course resembles our proposal to test personalized CBT, albeit without matching the optimized intervention to the profile of individual patients. The factorial design also provides a strong test of the relative contribution of specific vs. common factors, which is another advantage. The design proposed by Watkins et al. is currently being used in an ongoing large-scale RCT and the results are underway (106).

Some psychotherapists will say that personalization of therapy is their everyday work, so who needs such a research agenda? They will adapt the therapy to their individual patients based on what they feel is the right combination of therapeutic procedures, based on their clinical intuition. But as Meehl (107) already demonstrated in 1954 and as we recently confirmed (108), clinical intuition is an unreliable source of input for the clinician. Therapists have their own thoughts and preferences on why and how to deviate from treatment protocols to treat their patients best, but such choices are most often not substantiated by empirical evidence. In the worst scenario, it can become a case of “therapist-centered psychotherapy,” where therapists deliver an eclectic therapy that “feels” best to themselves mostly. We strongly urge psychotherapists to take a more empirical stance toward their profession.

Our main message centers around the personalized causal pathway hypothesis that emphasizes the distinction between procedures and processes and calls for moderated mediation analyses or other approaches that take individual differences into account. We described the type of research that we think will be needed to advance our understanding of the mechanisms of change in psychotherapy, acknowledging that there are more roads that lead to Rome. Holmes et al. (103) concluded that advances in this field will depend on funding opportunities and greater collaboration among clinical researchers to establish the sample sizes that are required for this kind of research. We agree with these authors and invite researchers to engage in multi-lab collaborations to pool large datasets that can be used explore questions about personalizing CBTs.

## Data Availability Statement

The original contributions generated for the study are included in the article/supplementary material, further inquiries can be directed to the corresponding author/s.

## Author Contributions

MH drafted a first version of the manuscript. LL-L, PC, and NK provided feedback and additions to the text. All authors contributed to the article and approved the submitted version.

## Conflict of Interest

The authors declare that the research was conducted in the absence of any commercial or financial relationships that could be construed as a potential conflict of interest.

## Footnotes

^1^In this earlier study, we used linear regression models for within-sample predictions, which might have led to statistical overfitting. Modern machine learning approaches are now used to produce more stable and generalizable prediction models (17).
